# A survey of nurse practitioner perceptions of integration into acute care organisations across one region in Ireland

**DOI:** 10.1111/jonm.13602

**Published:** 2022-03-26

**Authors:** Mary Ryder, Paul Gallagher

**Affiliations:** ^1^ School of Nursing, Midwifery & Health Systems University College Dublin Dublin; ^2^ Ireland East Hospital Group Dublin Ireland

**Keywords:** integration, leadership, nurse practitioners, nursing management

## Abstract

**Aim:**

The purpose of the study was to explore nurse practitioner perceptions of integration practices in acute hospitals across one health care region in Ireland.

**Background:**

A recent Department of Health National policy towards developing a critical mass of nurse practitioners was implemented across Ireland. Successful integration of nurse practitioner roles is integral to the success of the service and sustainability of the roles for the long term.

**Method:**

An electronic survey was circulated to a convenience sample of 85 nurse practitioners across a single, acute health care region in Ireland.

**Results:**

Sixty‐six (78%) of nurse practitioners participated. A standardized governance structure was reported by 24 (36%) participants. Thirty‐two (48%) participants expressed their job description clearly defined their role. Consultant physicians were identified as the most supportive stakeholder by participants.

**Conclusions:**

This research identifies that nurse practitioner integration is not currently structured. A framework to support nurse practitioner integration is required to ensure ongoing support for the role. This research identifies that integration is not currently optimized.

**Implications for Nursing Management:**

Failure to successfully integrate the nurse practitioner role risks the long‐term sustainability of the role and is a missed opportunity to demonstrate the success of advanced clinical leadership to health care.

## 1 INTRODUCTION

Nurse practitioners are individuals whom have extensive clinical experience and completed specialized education to a minimum level of master's degree (International Council of Nurses, 2020). In addition, nurse practitioners are authorized to practice autonomously and independently with agreed patient caseloads (Ryder et al., 2019). The nurse practitioner role has been associated with extensive research to date primarily describing work practices. Integration relates to the normalization of the nurse practitioner becoming embedded in‐service delivery and differs from nurse practitioner roles as pilot or test initiatives (Lowe et al., 2018). The integration of nurse practitioner roles depends on a structured, multilevel support system to ensure they are embedded within the service rather than an ‘add on’ (Contandriopoulos et al., 2015; Lowe et al., 2018). Recent evidence from Australia has emerged to suggest however that role integration is inconsistent, yet the success of the role and sustainability of the nurse practitioner service is dependent of successful integration (Fox et al., 2018; Lowe et al., 2018). No research has been completed to date exploring nurse practitioner integration from an Irish perspective.

## 2 BACKGROUND

The Irish health care system is undergoing radical change that is underpinned by Department of Health policy (Committee on the Future of Healthcare, 2017). In 2017, there was a national initiative in Ireland led by the Department of Health to create a critical mass of Registered Advanced Nurse Practitioners and Midwifery Practitioners in the health service (Department of Health, 2019). The objective of the initiative was to increase nurse practitioners in the health service, to achieve 2% of the nursing workforce, to work as nurse practitioners within a defined period of time. At that time, 120 new nurse practitioner positions were identified through a central business case application review process. The initiative included the development of a National Higher Education Consortium to deliver a standardized broad‐based education curriculum to candidate nurse practitioners. In addition, the initiative included a recommendation for health care organisations to develop local implementation groups (LIG) at hospital or group level.

An evaluation report, of this initiative (Brady et al., 2020), provided detailed descriptions of nurse practitioner activities and outcomes to health care. Many findings were consistent with previous nurse practitioner evaluations, both nationally and internationally (Begley et al., 2010; Gardner et al., 2010; Ryder et al., 2020).

The Brady et al. (2020) report also identified that one of the key barriers to the implementation of the policy and sustainability of the nurse practitioner role was organisational governance issues and standardized processes. This finding is also consistent with international literature having previously reported nurse practitioner experiencing resistance and isolation during the early stage of integrating into their respective organisations (Ryder et al., 2019).

These findings are not exclusive to Ireland; indeed, they are consistent with previous literature, identifying nurse practitioner having trouble integrating their roles into the clinical setting (Kilpatrick et al., 2019; Ryder et al., 2019). Lowe et al. (2018) provide a solution whereby organisational cultures and strong leadership influence the successful integration of nurse practitioner roles in organisations. Five factors were identified by Contandriopoulos et al. (2015) in a process for successful implementation of nurse practitioner roles in primary care settings. The factors identified were planning, role definition, practice model, collaboration and team support. These factors are consistent with those recently reported in a scoping review by Whitehead et al. (2022) that contribute to successful transition from student to nurse practitioner roles, independent of the clinical setting.

Acknowledging the varying health care nuances between countries, the factors related to integration were considered applicable by the researchers from an Irish context. There was anecdotal evidence to suggest that nurse practitioner integration was inconsistent in some organisations. With an increasing number of nurse practitioner appointments, one health care region expressed an interest to explore integration from the perspectives of nurse practitioners. There is no research to date on nurse practitioner integration in Ireland.

## 3 AIM

The purpose of this project is to identify the nurse practitioner understanding of the process of integration across one health care region in Ireland. The research questions are as follows:

- Identify nurse practitioner reported planning, governance and support structures
- Determine the process applied to integrating nurse practitioner roles
- Establish the existence of collaborative referral procedures related to the nurse practitioner roles
- Ascertain performance measurement processes for nurse practitioners.

## 4 METHODS

### 4.1 Sample

A convenience sample of nurse and midwife practitioners working in one health care region in Ireland were selected. The total population of registered and/or candidate/student nurse practitioners in the health care region was 126. Candidate/Student nurse practitioners were excluded from this research as they had not completed their job preparations. The health care region, one of seven regions in Ireland, has a workforce of almost 5000 nurses and midwives across 11 acute hospitals and serves a population of 1.1 million people.

### 4.2 Instrument

A survey was developed from Contandriopoulos et al. (2015) framework developed for nurse practitioner integration in primary. The factors identified in the framework were reviewed and adapted to develop a survey in the context of nursing and midwifery in Ireland. The factor ‘planning’ was expanded to include governance and incorporate Contandriopoulos et al. (2015) factor ‘supporting teams’ for the integration of the nurse practitioner role. The research team determined that the factor ‘patient care model’ was incorporated into planning, governance and role definition and consensus where the model of practice is defined at the point of agreeing the caseload for nurse practitioners. It was determined that ‘collaboration and referral’ were terms that were used in the Irish context in preference to ‘practice model’ and ‘collaboration’ described by Contandriopoulos et al. (2015). The research team agreed that a factor related to outcome and performance measurement was essential in the integration process for nurse practitioners as it provides clarity to all stakeholders related to role expectations.

A cloud‐based survey ‘Nurse Practitioner Integration’ was designed by the researchers, using Qualtrics® software (Data S1). The survey comprised of 45 questions across five categories including general demographics, planning/governance/support, role definition/consensus, collaboration/referral and outcome/performance measurement (available upon request). The survey was tested for face validity among five nurse practitioners working in other health care regions in Ireland. The five nurse practitioners were randomly selected from a list of graduates from one Irish University. No modifications were recommended to the survey following review. Reliability was tested during analysis.

### 4.3 Data collection

An email was sent to a list of all nurse practitioners from the Chief Director of Nursing (DON) and Midwifery for the health care region in August 2021. The email included an anonymous link and QR code to the online survey. A reminder email was sent every 2 weeks over an 8‐week period.

### 4.4 Data analysis

Quantitative data were exported directly from Qualtrics® to SPSS 24® for statistical analysis. Data were checked and cleaned. The researchers concluded that three questions related to caseloads were not clearly defined therefore the data provided was excluded in the analysis. Descriptive statistics were used to describe, compare and summarize participant responses. Chi‐square statistics were used to establish an association between categorical variables.

## 5 ETHICAL CONSIDERATIONS

A declaration of exemption from full ethical review was accepted by the University human research ethics committee (LS‐E‐21‐175‐Ryder). The survey was accessed using an anonymous link or QR code. A participant information letter was provided upon accessing the survey. Participants were required to select an option ‘consent to participate’ to proceed to the survey questions. The first question required participants to identify their registration or candidate status. Selection of candidate practitioner resulted in automatic exit from the remainder of the survey.

## 6 RESULTS

Data were analysed using descriptive and analytical testing. Given the assumption of content and face validity based on the development of the survey. There is preliminary evidence of reliability Cronbach's *α* = .720 for 13 variables, eliminating two referral pattern questions.

### 6.1 Demographics

One hundred and four participants accessed the survey. A total of 34 candidate/student nurse practitioners were identified and excluded from analysis. Four additional blank survey responses were eliminated. A total of 66 responses from eligible nurse practitioners were included for analysis. Table 1 identifies that the largest number of participants worked in emergency care (*n* = 17; 26%), followed by older person care (*n* = 15; 23%). The majority of participants came into post in 2019 (*n* = 15; 23%). Forty‐three (65%) of participants were appointed as nurse practitioners since 2018.

**TABLE 1:** Demographic specialist area and year appointed

Specialist area	Frequency (*n*)	Percent (%)	Year appointed
Acute medicine	5	7.6	2016–2020
Ambulatory care	1	1.5	2015
Cardiology	3	4.5	2015–2020
Dermatology	3	4.5	2018
Diabetes	2	3	2020–2021
Emergency	17	25.8	2004–2019
Epilepsy	1	1.5	2020
Gastroenterology	2	3	2015–2016
Hepatology	1	1.5	2021
Midwifery	1	1.5	2016
Neonatology	1	1.5	2014
Older person	15	22.7	2016–2021
Oncology	1	1.5	2021
Ophthalmology	1	1.5	2019
Postnatal maternal	1	1.5	2020
Respiratory	4	6.1	2015–2021
Rheumatology	4	6.1	2018–2019
Thoracic surgery	1	1.5	2017
Urodynamics	1	1.5	2018
Vascular wound care	1	1.5	2018
Total	66	100	

### 6.2 Planning, governance and support

Planning for the nurse practitioner role is described by Contandriopoulos et al. (2015) as a process to identify the intended practice of the nurse practitioner and creating a vision for the role. Participants reported the DON (*n* = 47; 71%) and the medical physician (Consultant) in specialist area (*n* = 43; 65%) as the key stakeholders who identified an opportunity for the nurse practitioner role (Table 2). Participants were asked if there was a standardized governance structure for the development and integration of nurse practitioner roles in their organisation. Twenty‐four (36%) participants reported having a standardized organisational governance structure. More than half (*n* = 36; 55%) provided an annual report primarily to the DON (*n* = 29; 44%) (Table 2).

**TABLE 2:** Stakeholder engagement with nurse practitioner/midwife practitioner governance procedures

	Key stakeholder identifying role	Annual report	Most significant support person	Contribute to job description	Contribute to performance indicators
	*n* (%)	*n* (%)	*n* (%)	*n* (%)	*n* (%)
Director of nursing/midwifery (DON/DOM)	47 (71)	29 (44)	8 (12)	25 (38)	13 (20)
Directorate nurse/midwife manager	17 (26)	5 (8)	2 (3)	6 (9)	4 (6)
Assistant director of nursing/midwifery	14 (21)	7 (11)	N/A	7 (11)	2 (3)
Nursing/midwifery practice development	13 (20)	5 (8)	3 (5)	12 (18)	7 (11)
Nursing and midwifery planning and development unit	19 (29)	0	2 (3)	10 (15)	4 (6)
Consultant in specialist area	43 (65)	18 (27)	31 (47)	33 (50)	13 (20)
Registered advanced nurse practitioner in similar area	N/A	N/A	7 (11)	27 (41)	8 (12)

Three questions focused on ongoing role review. One third of participants (*n* = 20; 30%) have an agreed process to discuss opportunities for expansion of their service. A similar number (*n* = 21; 31%) reported having their caseload (workload reviewed) at defined times, which varied from 3 to 72 months. Eight participants (12%) reported their organisation had an agreed process to review their role and workload. The largest proportion of participants (*n* = 31; 47%) identified the consultant physician as the most significant support person in their role (Table 2).

The majority (*n* = 41; 62%) reported the existence of a nurse practitioner network in their organisation. This was described as a professional supportive network, led primarily by nurse practitioners.

### 6.3 Role definition and consensus

This is defined as the process of defining and agreeing the nurse practitioner caseload within the area of practice (Contandriopoulos et al., 2015). Most participants (*n* = 45; 68%) reported having a specific agreed job description. Three key stakeholders were identified by participants as contributing to the job description, Consultants, DON, and a nurse practitioner in a similar role (Table 2). Thirty‐two participants (48%) expressed that their job description provided provide clear details about their role.

### 6.4 Collaboration and referral

The language collaboration and referral relate to the process of agreeing autonomous and collaborative caseloads and referral process for the nurse practitioner service. Most participants (*n* = 40; 61%) expressed that their job description specified work that was autonomous and collaborative in nature. The majority reported having agreed organisational referral processes to access their service (*n* = 41; 62%) and to refer to other services (*n* = 45; 68%). More than half of the participants reported having agreed external (*n* = 34; 51%) referral processes for their service.

### 6.5 Outcome and performance measurement

Outcomes demonstrate the value of a service and are a requirement in the Irish context (Department of Health, 2019). The process of defining outcome and performance measures provides clear expectations of the nurse practitioner role. Twenty‐five (38%) of participants reported having agreed performance indicators for their service. Of those that identified having performance indicators the stakeholder contribution to these was varied (Table 2). Fifteen (23%) participants identified their performance indicators aligned to the 2019 Department of Health policy document. A smaller number (*n* = 11; 17%) understood their performance indicators reflected access to their service and that they captured the quality of care provided. A small number of participants (*n* = 7; 11%) believed performance indicators accurately captured their workload. Reporting timelines for performance indicators have been identified for 12 (18%) participants.

### 6.6 Relationships

Chi‐square statistical analysis identified that having agreed performance indicators for nurse practitioner roles is dependent on having a standardized governance structure in an organisation; however, the relationship was not significant, χ^2^ (2, *N =* 46) = 4.889, *p* = .087.

### 6.7 Open comments

Participants were provided with an opportunity to provide any comments in the final question. Twelve participants expressed a need for additional support in both the establishment and meeting the ongoing requirements of their role.

## 7 DISCUSSION

There was a large response from nurse practitioners to the survey. This research has identified a recent increase in nurse practitioner roles in areas outside the Emergency Departments, focusing on older person services in response to a strategic centralized approach from the Department of Health. Identifying a gap in service can be best served by nurse practitioners was a joint effort by Directors of Nursing and Consultant physicians. Participants reported a lack of governance structures for these senior nursing clinical positions across the health care region. The nurse practitioners in this research reported rich collaborations and referral processes both within their own organisations and externally. Less than half of participants identified having performance indicators associated to their services. Fewer than one quarter reported their performance indicators were aligned to recent health care policy, and most participants expressed that they did not capture the nature of their work. Ongoing role and service review was reported as lacking by participants. Consultant physicians were identified as the key stakeholder supporting nurse practitioners in their roles.

Support from policy makers is essential to the development of nurse practitioner initiatives (Lowe et al., 2018). The Department of Health in Ireland developed a policy to support the development of a critical mass of nurse practitioner roles in targeted areas (Department of Health, 2019). The impact of this policy in supporting the development of nurse practitioner roles is evident in the large number of roles since 2018, particularly in older person services (Department of Health, 2019). An increase of nurse practitioners in older person services is an international trend coupled with worldwide increase in seniors requiring long‐term care (Kilpatrick et al., 2019). Despite the widely acknowledged improvement in evidence‐based practice and quality of care associated with nurse practitioner care, there are consistent reports of barriers towards successful integration of the role (Kilpatrick et al., 2019; Lowe et al., 2018; Ryder et al., 2020; Torrens et al., 2020). The literature enforces the importance of integration for nurse practitioners identifying that successful integration is critical to the long‐term sustainability of the role (Marceau et al., 2021).

### 7.1 Planning governance and support

This research suggests a dependence on DONs and Consultants to create a vision and planning for the nurse practitioner role, contribute to defining the role, and determine outcome and performance measures. This research suggests this occurs in the absence of a broader collaborative approach with other nursing managers with operational responsibilities for areas where nurse practitioners will be working. The findings also suggest that the expertise of nursing planning and development departments are not included in factors related to nurse practitioner role integration. The levels of support from stakeholders, within organisations, perceived by nurse practitioners in this research was mixed, which is consistent with similar findings previously reported in the literature (Clifford et al., 2020; Ryder et al., 2019). Consistent with the findings of this research, Boman et al. (2019) reported that stakeholder and management support is required at the development and integration stages of the nurse practitioner role. Resonant with findings from Lowe et al. (2018), successful integration requires supporting a change management process facilitated by management. The evidence suggests that the lack of support perceived by nurse practitioners results in feelings of isolation for nurse practitioners who are often left to negotiate and explain the purpose of their role themselves (Ryder et al., 2019). Participants in this research cited the consultant physician as the most significant support person in their role. This is consistent with previous findings in Ireland that reported consultant physicians were supportive of nurse practitioner roles, particularly in their research role (Ryder et al., 2020).

### 7.2 Role definition and consensus

Participants reported having a specific job description; however, the majority expressed this did not provide a clear description of their service. This research suggests that there may be opportunities for greater collaboration, or delegation within senior nursing management and experts in planning and development towards a collaborative, supportive, integrative framework for nurse practitioners. Applying a collaborative structured process to nurse practitioner integration in health care organisations provides an opportunity to address the specific characteristics required to support successful integration in clinical practice settings. Lack of clearly defined roles and responsibilities in health care teams has been identified as an obstacle in multi‐professional collaborative health care delivery that is the hallmark of patient care delivery (Sørensen et al., 2020). This also raises the question as to whether this is a missed opportunity for nursing management to provide equivalent supports to ensure the role meets the nursing strategies in organisations.

### 7.3 Collaboration and referral

Researchers in Canada (Mian et al., 2012) have previously reported bidirectional referral patterns between nurse practitioners, general practitioners (GP) and allied health care professionals (AHP) indicate successful integration of nurse practitioner roles in health care. The findings in this research identified a high level of referral patterns related to health care services provided by nurse practitioners both interprofessional within organisations and among the wider community health care structures. There is a dearth of literature related to nurse practitioner referral patterns nationally and internationally. The only Irish research related to nurse practitioner referral (Ingram et al., 2017) reporting on the efficiency of referral from ED to a specialized chest pain clinic in a single centre. Comparing the outcomes of nurse practitioner referrals to their health care colleagues are also reported in other specialist areas (Gelinne et al., 2019; Liddy et al., 2016). This would reflect positively on nurse practitioner integration among the participants in this study.

### 7.4 Outcomes and performance measurements

Participants in this research indicated that performance measurement and outcome is not widely measured or captured. This is arguably accurate considering the number of nurse practitioners internationally and acknowledging there is a paucity of evidence reporting improved patient outcomes (Ryder et al., 2020; Smigorowsky et al., 2019). There are some single centre nurse practitioner service evaluations reported in the literature (Ryder et al., 2020). A multisite exploration of patient indicators in emergency departments indicated that the nurse practitioner role was associated with lower performance for service indicators in Australia (Middleton et al., 2019). This research suggests a lack of attention to performance and outcome measurements is evident.

This research reports the absence of a consistent structured process in the regional health care area to support the successful integration of these clinical leaders into health care organisations. The findings in this research are consistent with international literature reporting the lack of consistency towards the integration of nurse practitioners in health care services and are described as ad‐hoc (Adams & Carryer, 2019). The process to successfully integrate nurse practitioners into health care organisational structures has been descried as a change management process by Lowe et al. (2018).

This was the first research in Ireland to explore nurse practitioner integration. The research captures the impact that recent national health care policy has successfully achieved a critical mass of nurse practitioner roles focusing on key areas, particularly older person services. The long‐term success of the nurse practitioner roles lies in the successful integration into health care organisations, which includes planning, governance, support, collaboration and outcome reporting. The research is timely to address the gaps evident in nurse practitioner integration that are highlighted by participants in this research.

## 8 CONCLUSIONS

This study is the first in Ireland to examine the perceived integration of nurse practitioners in health care organisations across a regional health care area. The study has provided evidence that a national policy developed to create a critical mass of nurse practitioners in designated areas has achieved this target. The findings indicate an urgent need for a collaborative supportive process to agree essential characteristics related to nurse practitioner role integration, namely, governance, role clarity, collaboration, referral, and development and agreed outcome measurements. While the health service responds to the requirement for additional nurse practitioners, nursing management must use this as an opportunity to optimize the benefits that can be achieved from this clinically expert group to ensure the role of the nurse practitioner meets the strategic nursing and organisational requirements.

## 9 IMPLICATIONS FOR NURSING MANAGEMENT

The findings in this research identify that integration of nurse practitioner roles across a large health care region in Ireland is inconsistent and lacking in structure. Nurse managers and nurse leaders in Ireland must respond to the radical change in progress in the Irish health care system. The role of nursing management in the context of enabling and supporting nurse practitioners to fulfil their role has never been as important.

## CONFLICT OF INTEREST

There are no conflicts of interests to report in this research.

## ETHICS STATEMENT

A review of a declaration of exemption from full ethical review was accepted by the University Research Ethics Committee. Participation information was provided at the beginning of the survey. Participants were informed that completion of the survey implied consent (LS‐E‐21‐175‐Ryder).

## Supporting information


**Data S1.** Supporting Information

## Data Availability

Research data are not shared.
