# Supplementary material for: A survey of nurse practitioner perceptions of integration into acute care organisations across one region in Ireland
Source: J Nurs Manag. 2022 Mar 26;30(4):1053–60. doi: 10.1111/jonm.13602 (PMC9314621; doi:10.1111/jonm.13602)
Supplement: Supplementary file 1 — Data S1. Supporting Information [file JONM-30-1053-s001.pdf]

Participant Information

We welcome your consideration to participate in this survey to establish the current process leading to the integration of RANP roles into organisations within the Ireland East Hospital Group (IEHG). This project is led by Mr Paul Gallagher, Chief Director of Nursing (IEHG) in collaboration with Dr Mary Ryder (UCD).

The recent Department of Health report of the Impact of Implementing a Draft Policy to Develop Advanced Nurse Practitioners (cANPs/RANPs) to Meet Health Service Needs (Brady et al 2021) identified that clarity was required to identify the process for the successful integration of RANPs into acute care organisations in Ireland. These findings are consistent with previous literature, including Irish research identifying ANP experiencing difficulty integrating their roles into the clinical setting (Ryder et al 2019). Lowe et al (2018) reported that organisational cultures and strong leadership influence the successful integration of ANP roles in organisations. Contandriopoulos et al (2015) has developed a framework as a guide to the successful integration of ANP roles in primary care. However, there is no framework to guide ANP integration in acute care organisations.

This online survey will take you approximately 10 minutes to complete. Your involvement in the research is entirely voluntary and anonymous. You may choose to leave any questions unanswered. You are under no obligation to complete the survey. There are no right or wrong answers; just the way you see the answers based on your current experience. The declaration for exemption from full ethical review has been approved by UCD Research Ethics Committee. Therefore, I would ask that you please take a few minutes and complete this survey now. If you wish to proceed, please select the button “I wish to participate in this survey”.

Survey Questions

General demographic questions

1. I am currently

    a. A candidate ANP/AMP (no need to continue)
    b. A Registered ANP/AMP

2. I am in post as an RANP/RAMP since: (state year)

3. My clinical specialist area is: (state)

Planning/ Governance/Support

4. Can you please identify the key stakeholders that worked toward identifying an opportunity for an ANP/AMP in your service? (Please select all that apply)

    a. Director of Nursing/Midwifery
    b. Directorate Nurse/Midwife Manager
    c. Assistant Director of Nursing/Midwifery
    d. Nursing/Midwifery practice Development
    e. Nursing and Midwifery Planning and Development Unit
    f. National Clinical Care Programme Initiative
    g. Consultant in Specialist Area

5. There is an ANP/AMP professional network in my organisation (A professional network is a group of people who have connected with one another for career or business-related reasons).

    a. Yes
    b. No (please proceed to question 8)

6. The ANP/AMP network is led by

    a. N/A
    b. DON/DPM or designate (e.g., ADON/ADOM/Directorate Nurse Manager)

c. ANP/AMP
d. Other (state)

7. Can you describe the purpose of the ANP/AMP network in your organisation? (Open text)

8. Is there a standardised governance structure for the development and integration of ANP/AMP roles in your organisation?

   a. Yes
   b. No
   c. Don’t Know

9. Can you please outline your reporting structures for your role in your organisation?

   a. Professional reporting to (Please state)
   b. Clinical reporting to (Please state)
   c. Operational reporting (Please state)

10. Do you provide an annual report?

   a. Yes
   b. No

11. Whom do you send an annual report to?

   a. Director of Nursing/Midwifery
   b. Assistant Director of Nursing/Midwifery
   c. Nursing/Midwifery Practice Development
   d. NMPDU
   e. Consultant
   f. Department
   g. I do not write an annual report

12. Is there an agreed process in your organisation to discuss opportunities for expansion of your RANP/RAMP service?

a. Yes
b. No
c. Don't know

13. Can you list the stakeholders that would be included in discussions related to expansion of your RANP/RAMP service?
(Please list all that apply)

14. Who would you describe as the most significant support person in your RANP position? (Please select only one)

   a. Director of Nursing/Midwifery
   b. Directorate nurse Manager
   c. Assistant Director of Nursing/Midwifery
   d. Nursing/Midwifery Practice Development
   e. NMPDU
   f. Consultant
   g. Other (Please identify one professional)

15. Can you identify any additional supports that you have identified that have contributed to the successful integration of your RANP/RAMP role in the organisation?
(Open text)

Role Definition and Consensus

16. I have a job description specific to my RANP/RAMP role?

   a. Yes
   b. No
   c. Don't Know

17. Was there a consensus on your specific role when you completed your registration?

a. Yes
b. No
c. Don't Know

18. Can you list the persons that contributed to your specific job description? (Please list all that apply)

    a. Director of Nursing/Midwifery
    b. Directorate Nurse Manager
    c. Assistant Director of Nursing/Midwifery
    d. Nursing/Midwifery Practice Development department
    e. NMPDU project officer
    f. Consultant in specialist area
    g. RANP/RAMP in similar role
    h. Other RANP/RAMP in the organisation
    i. Other (please specify)

19. Is there an agreed process for the review of your RANP/RAMP role and job description?

    a. Yes
    b. No
    c. Don't Know

20. How frequently is your role/job description amended to reflect a change in service? (Time in months)

21. In your opinion does your job description provide clear details about your RANP/RAMP role?

    a. Yes
    b. No
    c. Don't know

22. Do you have a number of defined caseloads or your RANP/RAMP role?
(caseload is the definition of the number and types of patients that you see in your role)

    a. Yes
    b. No (please proceed to question 26)

23. How many different caseloads do you manage in your RANP/RAMP role? (number)

24. Are your RANP/RAMP caseloads reviewed at defined times?

a. Yes (please go to Question 26)
b. No

25. Please state the frequency of caseload review for your RANP/RAMP role? (Time in Months)

Collaboration/Referral

26. Does your role/caseload specify aspects of your RANP/RAMP service are autonomously/collaboratively?

    a. Yes
    b. No

27. Does your caseload include internal referral processes to access your RANP/RAMP service?

    a. Yes
    b. No

28. Does your caseload include external referral processes to access your RANP/RAMP service?

    a. Yes
    b. No

29. Please identify the referral pathways to access your service (Select all that apply)

    a. Any Consultant
    b. Identified Consultant (please describe)
    c. Any NCHD
    d. Identified NCHD (please describe)

e. Any GP
f. Specified GP (please describe)
g. Public Health Nurses
h. Primary Care Nurses
i. Any RANP
j. Identified RANP (please describe)
k. Any CNS
l. Identified CNS (please describe)
m. CNM
n. Staff Nurse/Midwife
o. Any allied healthcare professional
p. Specified allied health professional (please describe)
q. Via specified pathway
r. other

30. Does your caseload include internal referral processes from your RANP/RAMP service to other services (including but not limited to physiotherapy etc)?

    a. Yes
    b. No

31. Does your caseload include external referral processes from your RANP/RAMP service to other services?

    a. Yes
    b. No

32. Please list other services that you refer your patients to.
    (Please list all that apply)

33. Can you briefly describe the interface between your service and the community? (Open text)

Outcome/Performance Measurement

34. Do you have agreed performance Indicators (KPI)?

a. Yes
b. No (You have successfully completed the survey, thank you for your contribution)

35. Please identify the personnel that were included in the decisions related to your performance indicators (please select all that apply)

    a. Director of Nursing/Midwifery
    b. Directorate Nurse Manager
    c. Assistant Director of Nursing/Midwifery
    d. Nursing/Midwifery Practice Development department
    e. NMPDU project officer
    f. Consultant in specialist area
    g. RANP/RAMP in similar role
    h. Other RANP/RAMP in the organisation
    i. Other (please specify)

36. How many performance indicators do you have?
    (Number)

37. Are your performance indicators aligned to the recommendations in the Department of Health policy document (2019)?

    a. Yes
    b. No
    c. Don't know

38. In your opinion do your performance indicators reflect access to your RANP/RAMP service?

    a. Yes
    b. No

39. In your opinion do your performance indicators capture the quality of care provided by your RANP/RAMP service?

    a. Yes

b. No

40. If Yes, what kind of quality indicators (e.g. patient experience; Patient reported Outcomes /PROM's)?
    (Please list all that apply)

41. Do you feel that your performance indicators capture your workload accurately?

    a. Yes
    b. No (please describe why)

42. Do your performance indicators identify reporting timelines?

    a. Yes
    b. No (please proceed to question 20)

43. Please describe your reporting timelines (for KPI’s)
    (Open text)

44. In your opinion, what value do performance indicators add to your role? (Open Text)

Contandriopoulos, D., Brousselle, A., Dubois, C., Perroux, M., Beaulieu, M., Brault, I., Kilpatrick, K., D'Amour, D., & Sansgter-Gormley, E. (2015). A process-based framework to guide nurse practitioners integration into primary healthcare teams: Results from a logic analysis. *BMC Health Services Research, 15*(1), 78-78. https://doi.org/10.1186/s12913-015-0731-5
